# A systematic review and meta-analysis of the effectiveness of virtual reality as an exercise intervention for individuals with a respiratory condition

**DOI:** 10.1186/s41077-020-00151-z

**Published:** 2020-11-19

**Authors:** Christina Condon, Wing Tung Lam, Chiara Mosley, Suzanne Gough

**Affiliations:** 1 Faculty of Health Sciences and Medicine, Bond University, Gold Coast, Queensland Australia; 2 Faculty of Health, Psychology and Social Care, Manchester Metropolitan University, Manchester, UK

**Keywords:** Virtual reality, Virtual reality system, Exercise, Exergaming, Gaming, Intervention, Mixed reality, Augmented reality, Rehabilitation, Respiratory

## Abstract

**Background:**

Respiratory diseases impose an immense health burden worldwide and affect millions of people on a global scale. Reduction of exercise tolerance poses a huge health issue affecting patients with a respiratory condition, which is caused by skeletal muscle dysfunction and weakness and by lung function impairment. Virtual reality systems are emerging technologies that have drawn scientists’ attention to its potential benefit for rehabilitation.

**Methods:**

A systematic review and meta-analysis following the PRISMA guidelines was performed to explore the effectiveness of virtual reality gaming and exergaming-based interventions on individuals with respiratory conditions.

**Results:**

Differences between the virtual reality intervention and traditional exercise rehabilitation revealed weak to insignificant effect size for mean heart rate (standardized mean difference, SMD = 0.17; *p* = 0.002), peak heart rate (SMD = 0.36; *p* = 0.27), dyspnea (SMD = 0.32; *p* = 0.13), and oxygen saturation SpO_2_ (SMD = 0.26; *p* = 0.096). In addition, other measures were collected, however, to the heterogeneity of reporting, could not be included in the meta-analysis. These included adherence, enjoyment, and drop-out rates.

**Conclusions:**

The use of VRS as an intervention can provide options for rehabilitation, given their moderate effect for dyspnea and equivalent to weak effect for mean and maximum peak HR and SpO_2_. However, the use of virtual reality systems, as an intervention, needs further study since the literature lacks standardized methods to accurately analyze the effects of virtual reality for individuals with respiratory conditions, especially for duration, virtual reality system type, adherence, adverse effects, feasibility, enjoyment, and quality of life.

## Background

Respiratory diseases impose an immense health burden worldwide and affect millions of people on a global scale [1]. Chronic obstructive pulmonary disease (COPD), lung cancer, acute respiratory infections, tuberculosis, and asthma are the five most common respiratory conditions [1]. The affected population commonly experience symptoms including coughing, excessive sputum production, and shortness of breath [1], as well as other repercussions including reduced quality of life, systemic inflammation, decreased exercise tolerance, deconditioning, and inactivity [2]. Reduction of exercise tolerance poses a huge health issue affecting the cystic fibrosis (CF) patients, which is caused by the skeletal muscle dysfunction and weakness and lung function impairment resulting from CF [3, 4].

Virtual reality (VR) is an emerging new technology that has drawn scientists’ attention to its potential impact on rehabilitation. The American College of Sports Medicine identified that modern technologies, including virtual reality, are the upcoming trend for rehabilitation and promoting an active lifestyle [5]. Based on their systematic review, Butler and colleagues conclude that active videogames induce similar physiological demands, such as maximal heart rate, dyspnea levels, and energy expenditure during training as traditional exercise modalities [6]. Obtaining clinical control, particularly in chronic respiratory conditions, can have systemic effects for the patient from length of hospital stay to quality of life. Gomes et al. recognized a key pillar of clinical control in pediatric populations with asthma and further concluded that rehabilitation utilizing VR exergaming was beneficial in improving cardiorespiratory fitness and symptoms relief in children with asthma [7]. Almeida and Rodrigues provided statistically significant evidence advocating for the implementation of VR in pulmonary rehabilitation program by highlighting its benefits on symptom relief, improved health-related quality of life, shorter duration of hospitalization, and reduction of healthcare cost [8].

Compliance is a pivotal factor influencing the effects of the rehabilitation program, especially for chronic disease. Unfortunately, poor patient compliance is a common issue, observed among studies and it is associated with frequent exacerbations of symptoms and more hospital admissions [9, 10]. Among asthma patients, fear of exacerbation contributed to non-compliance and poor participation rate of physical activities, although research has clearly pointed out the unlikelihood of such adverse events during exercise [10]. Burr et al. suggested a clinical guideline to further assist the physicians to develop exercise guidelines for their clients, based on their condition and personal goals, with only 26 (3.4%) out of 770 pre-screened participants reported the occurrence of mild adverse events [10]. The potential benefits of physical activity outweigh the risks and a strategy to promote compliance can be the key to respiratory rehabilitation. Home-based exercise training programs provide an opportunity for patients with CF to continue engaging in physical activities after they are discharged from hospitals, where they were constantly under supervision. However, observational studies involving home-based exercise programs [9] and clinical guidelines [10] both revealed inconsistent results in adherence which potentially led to unsatisfactory outcomes.

Exercise programs incorporating video game activities (VGA) provide an alternative to pulmonary rehabilitation programs. Besides the health benefits, VGA has the capability to affect enjoyment, adherence, and motivation to physical activities, especially in the young population [6]. Virtual reality gaming (VRG) is reported to be preferable to traditional exercise in CF and COPD studies because it is both enjoyable and can easily be implemented in their daily life [11, 12]. One of the challenges incorporating VR into a rehabilitation program is to achieve high training loads needed to guarantee training effects [11]. Rutkowski et al. examined the effect of virtual reality systems (VRS), which is an umbrella term for virtual reality (VR), virtual reality gaming (VRG), augmented reality (AR), and mixed reality (MR). They highlighted the similarities between exercise incorporated VRS and traditional rehabilitation exercise in body movements [12]. Other studies supported the previous claim and proved rehabilitation programs utilized VRS elicit similar physiological outcomes, such as improved exercise capacity and responses including heart rate [13]. VRS are developed with the purpose to engage users by creating interactive and stimulating environments via visual, audio, and/or hepatic stimulus [14]; by encouraging engagement, VRS have a promising potential to increase motivation and compliance to exercise programs [15, 16].

The feasibility of using exergaming-based intervention as an alternative to traditional exercise intervention is still ambiguous with limited unbiased research conducted. Finite evidence was provided on feasibility and adverse events [16]. Most of the studies were confined by their small sample size and the preselection of relatively healthy participants [6, 9, 12, 13]. Due to the dynamic nature of this field, it is imperative that the literature targets the most recent findings. The current systematic review aims to provide a unique exploration beyond the scope of previous reviews, which have primarily focused on active video games integrated within the treatment of chronic respiratory diseases, cystic fibrosis, or obstructive respiratory conditions. The purpose of this systematic review is to investigate the most current literature that examines the effectiveness of VR gaming and exergaming-based interventions in individuals with a respiratory condition and to provide further direction and recommendations toward future research.

## Methods

A systematic review and meta-analysis following the PRISMA guidelines was performed to explore the effectiveness of VRS gaming and exergaming-based interventions on individuals with respiratory conditions.

### Search strategy

From September 2019 to November 2019, databases were systematically searched: PubMed, Cumulative Index to Nursing and Allied Health Literature (CINAHL), Embase, Medline, Web of Science, and Cochrane library. After an initial search with mutually agreed search terms, an extensive search strategy was created, and search terms were individualized for each database with results being entered into a reference management tool (Endnote v9). An additional search using all identified terms and index words was done. The search terms used can be placed in three categories: condition, virtual reality, and gaming (Table 1). Reference lists were researched and collated manually and independently by two reviewers (CC and WL). Manual searches of the gray literature revealed no additional relevant results.

**Table 1:** Search terms

Condition	Virtual reality	Gaming
Pulmonary disease, chronic obstructive	Virtual reality	Exergaming
Respiratory condition	Virtual reality exposure therapy	Video games
Respiratory tract diseases	Augmented reality	Gaming
Restrictive pulmonary disease	Mixed reality	Game
Chronic obstructive pulmonary disease		PlayStation
Lung cancer		Wii
COPD		Nintendo
Chronic bronchitis		Kinect
Emphysema		Xbox
Asbestosis		Interactive games
Asthma		
Cystic fibrosis		
CF		
Bronchiectasis		

### Study selection

Two reviewers (CC and WL) independently searched the databases systematically to identify relevant articles. Relevant results were entered into a reference management tool (Endnote v9) and duplicates were removed. Eligibility screening of articles was done independently by the reviewers. Additionally, articles that met the inclusion criteria were screened for further eligible studies. Conference abstracts as well as those where full text was not available were removed. The two authors compared studies for inclusion and exclusion. A third author (SG or CM) resolved discrepancies in decision-making. No language restrictions were applied to the search; however, all search results were written in English.

**Fig. 1:**
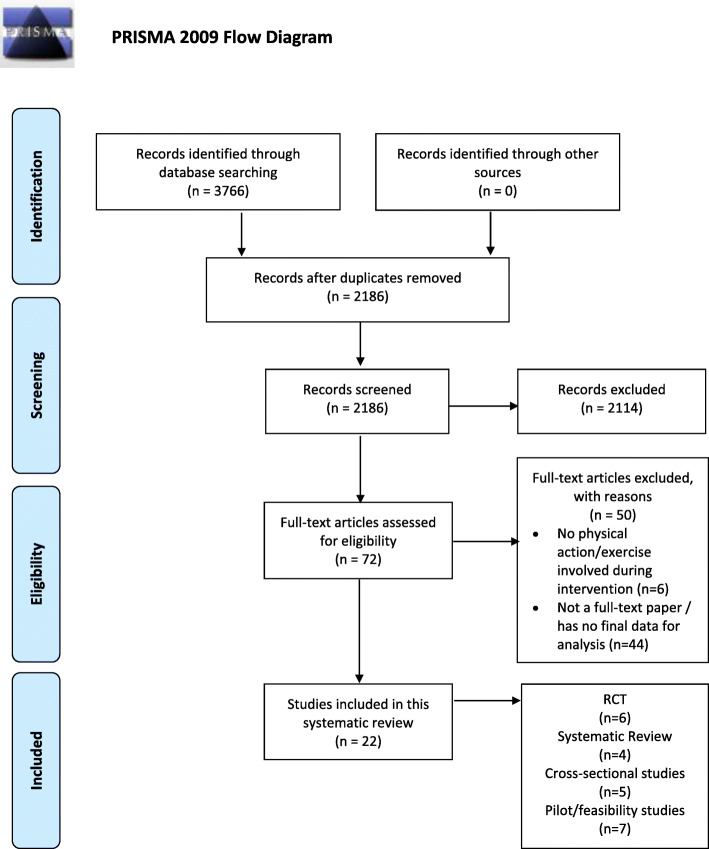
PRISMA flow diagram for article inclusion

Overall, 3766 articles were identified after the process of literature search utilizing the search strategy. After an initial abstract and title screening by two independent reviewers (CC and WL), 72 articles were deemed relevant and eligible. All 72 full-text articles were subjected to the inclusion and exclusion criteria (Table 2). Twenty-two relevant articles were selected from 3766 potential papers using the PRISMA process (Fig. 1). Seven articles were identified as pilot or feasibility studies, while the remaining articles were either systematic reviews (SR) (*n* = 4), randomized controlled trials (RCTs) (*n* = 6), or observational studies (*n* = 5). The Joanna Briggs Institute (JBI) critical appraisal tools were used to evaluate quantitative and quality evidence [17, 18] of the included studies.

**Table 2:** Inclusion and exclusion criteria

Inclusion criteria	Exclusion criteria
Quantitative, qualitative, mixed method, narrative, case control and descriptive studies, randomized and non-randomized control trials, quasi-randomized trials, and case reports and surveys are all considered.	Interventions that are not requiring participants to perform any physical actions during the game, for example, educational games.
Participants are individuals with a clinical diagnosis of respiratory conditions. Respiratory conditions include chronic respiratory diseases (asthma, bronchiectasis, chronic obstructive pulmonary disease (COPD), cystic fibrosis (CF), bronchiectasis, restrictive pulmonary disease, lung cancer)	Article that is not a full-text paper (e.g., thesis, conference abstract) or has no final data to be further analyzed.
Studies include an intervention involving any form of virtual reality (VR) gaming, augmented reality gaming, mixed reality gaming, exergaming, video game, console game, or game-based interventions that require players to perform physical exercise that becomes part of their rehabilitation.	Article that is not published in English.

### Data extraction

Tables were used to adduce data extracted from included studies; authorship, geographic region, research type, population statistics, condition, duration, intervention type and comparator (Table 3), and outcome measures, adverse effects, limitations, and findings (Table 4) were recorded. The data extraction was completed by two reviewers (CC and WL).

**Table 3:** Overview of selected studies and their characteristics

Authors (year) country [reference]	Study type	Characteristics of participants			Duration	Intervention characteristics G: Game type E: Exercise type C: Console enhancement F: Frequency I: Intensity T: Time	Comparator(s)
		***N***	Age ± SD and gender	Condition			
Del Corral et al. (2018) Spain [19]	RCT	40 (20 intervention and 20 control)	Intervention: 12.6 ± 3.4 years old, 10 m/10f Control: 11 ± 3 years old, 11 m/9f	Cystic fibrosis	6 weeks 12 months	G: Routine mx and normal exercise routine and Wii Fit Plus E: Full body C: Wii Fit balance board F: 5 days/week I: 70-80% MHR T: 30-60 min Routine mx and normal exercise routine and home gaming program as above G: Wii Fit F: 2 days/week I: 70-80% MHR T: 20 min	Routine management and normal exercise routine Routine management and normal exercise routine
Gomes et al. (2015) Brazil [7]	RCT	36 (20 intervention and 16 Control)	Intervention: 7.5 ± 1.9yo, 7 m / 13f Control: 8 ± 2 years old, 7 m/9f	Asthma	8 weeks	G: Xbox Kinect “Reflex Ridge” E: Full body C: Virtual trainer F: 2 days/week I: Incremental increase (mean 90.5% MHR) T: 10 × 3 min, 30 s rest in between	F: 2 days/week I: Incremental increase (mean 65.2% MHR) T: Treadmill T: 30mins
Sutanto et al. (2019) Italy [20]	RCT	20 (10 intervention & 10 control)	Intervention: 65.1 ± 7.5 years old, 9 m/1f Control: 65.6 ± 4.7 years old, 10 m/0f	COPD	6 weeks	G: Wii Fit “Torso Twist” “Balance board,” “Free run” “Balance games”* and “Cycle”* E: Full body, lower limb only* C: Wii remote, balance board, and cycle F: 3 days/week I: Modified Borg scale: 5 T: 30 min each	F: 3 days/week I: Modified Borg scale: 5 T: Cycle T: 30mins
Kuys et al. (2011) Australia [21]	Randomized Cross-over study	19	28 ± 7 years old, 10 m/9f	Cystic fibrosis	2 days	G: Wii Active E: Full body C: Arm and leg straps motion detection F: 1 session I: Borg scale: 3-5 T: 15 min	F: 1 session I: Borg scale: 3-5 T: Treadmill/cycle T: 15mins
LeGear et al. (2016) Canada [22]	Randomized Cross-over study	10	65 ± 8.7 years old, 5 m/5f	COPD	1 day	G: Wii Active F: 1 session I: Borg scale: 3-5/RPE: 14-16 T: 152009min	F: 1 session I: Borg scale: 3-5/RPE: 14-16 T: Treadmill T: 15mins
Salonini et al. (2015) Italy [23]	Randomized Cross-over study	30	12 ± 2.5 years old, 11 m/19f	Cystic fibrosis	3 days	G: Xbox Kinect Adventures “River Rush” E: Full body C: Virtual trainer F: 1 session I: Game pre-set difficulty level (easy, intermediate, and difficult) T: 6 min × 3, 1 min rest in between	F: 1 session I: 80% of MHR T: Cycle T: 20mins
de Corral et al. (2014) Spain [11]	Observational cross-sectional study	24	12.6 ± 3.7 years old, 16 m/8f	Cystic fibrosis	Unclear	G: Wii Fit Plus/Wii Active/Wii Family Trainer E: Full body C: Wii balance board F: 2 sessions I: NA T: 5 min each, 30 min rest in between	6MWT
Frade et al. (2019) Brazil [24]	Observational cross-sectional study	50	66.7 ± 7.2 years old, 31 m/19f	COPD	1 week	G: Stationary avatar Walk “Gesture Maps” with VR, Xbox Kinect E: Full body F: 2 sessions I: NA T: 6 min	6MWT
Holmes et al. (2013) Australia [9]	Observational cross-sectional study	10	29 ± 6 years old, 6 m/4f	Cystic fibrosis	10 days	G: Xbox Kinect “Your Shape Fitness Evolved” E: Full body C: Virtual trainer F: 1 session I: NA T: 20 min	CPET
Liu et al. (2016) Netherlands [17]	Observational, cross-sectional study	109 (61 intervention +48 healthy control)	Intervention: 61.9 ± 6.8 years old, 38 m/23f Control: 61.6 ± 6.1 years old, 22 m/26f	COPD	2 days	G: GRAIL 6MWT E: Full body C: VICON motion analysis system F: 2 session I: NA T: 45 min	6MWT
O’Donovan et al. (2014) Ireland [25]	Observational, cross-sectional study	60 (30 intervention and 30 healthy control)	Intervention: 12.3 ± 2.6 years old, 17 m/13f Control: 12.2 ± 2.7 years old, 17 m/13f	Cystic fibrosis	1 day	F: 1 session I: NA G: Wii Sports Boxing and Wii Fit Jogging E: Full body C: Wii remote controller T: 15 min each with 5 min rest in between	6MWT
Butler et al. (2019) Canada [6]	Systematic review	6 studies	NA	Chronic respiratory diseases	NA	G: Xbox “Kinect Adventures” and Wii Fit “Jogging, Boxing & Dancing” E: Full body C: Wii Balance board, Virtual trainer	Treadmill, cycle, pulmonary rehabilitation
Carbonera et al. (2016) Brazil [26]	Systematic review	5 studies	NA	Cystic fibrosis	NA	G: Xbox Kinect “Your Shape Fitness Evolved” and “River Rush” and Wii Fit Free/Fit Plus/EA Sports Active/Family Trainer Extreme challenge E: Full body C: Wii Fit balance board, virtual trainer	Cycle, 6MWT, cardiopulmonary exercise test
Simmich et al. (2019) Australia [13]	Systematic review, meta-analysis	12 studies	NA	Respiratory conditions	NA	G: Xbox Kinect, Nintendo Wii and PC customized spirometer game E: Full body and respiratory exercises	CPET, incremental shuttle walk test, 6MWT, rest only
Sánchez et al. (2019) Spain [15]	Systematic review	9 studies	NA	Obstructive respiratory disease	NA	G: Wii Active, Xbox Kinect Adventure, Wii Fit, PC “The Asthma Files,” Super Nintendo “Bronkie’s Asthma Adventure” PC “Magic School Bus,” “Wee Willie Wheezie,” PC “WDTA” pediatric self-management of asthma game, PC Asthma Control, and PC “Asthma Command” E: Full body, education, and respiratory exercise C: Computer or game console, Wii controller, Wii Balance Board Spirometer	Routine treatment, treadmill, pulmonary rehabilitation, asthma information booklet, irrelevant Super Nintendo game, non-educational PC games, verbal asthma management plan

Abbreviations: *MHR* maximal heart rate, *RPE* rating of perceived exertion, *CPET* cardiopulmonary exercise test, *6MWT* 6-min walking test, *PC* personal computer, *WDTA* watch discover, think and act

*Target lower limb only

**Table 4:** Outcome measures and findings

Authors, (year) [reference]	Outcome measures	Adverse effect(s)	Limitation	Findings (mean ± SD)
Butler, Lee, Goldstein and Brooks (2019) [6]	HR, energy expenditure, dyspnea, health-related QoL	Not stated	Small number of studies with small sample size Risk of bias as hard to blind the participants	4 studies used HR as an outcome measure. 3 showed significant improvement after intervention and 1 showed higher MHR compared to CG. 1 study showed no differences in HR comparing VRG to CG 4 studies used energy expenditure as an outcome measure. 1 showed significant improvement after intervention and 1 study showed no differences in energy expenditure comparing VRG to CG 5 studies used dyspnea score as outcome measure. 3 showed significant improvement after intervention and compared to CG. 1 study showed no differences in energy expenditure comparing VRG to CG 2 studies measured health-related QoL as an outcome measure and they all showed improvement after intervention
Carbonera, Vendrusculo, and Donadio (2016) [26]	Primary: HR, Vo2 Secondary: Dyspnea and fatigue, SpO_2_, and energy expenditure	No adverse event	Small number of studies Great variance of type of exercise or test used as comparator Majority of studies used estimation of MHR, only one used objective measurement	No significant between-group difference in HR was found in 75% (*n* = 3) of included studies that compared HR between group (*n* = 4) VRGs achieved % of MHR recommended for training in 75% (*n* = 4) of the relevant included studies All VRGs achieved higher energy expenditure than the CGs in the relevant included studies (*n* = 2, 100%) Relevant included studies demonstrated a similar (*n* = 3, 75%) or higher (*n* = 1, 25%) between group SpO_2_ measurements Relevant included studies showed a lower level (*n* = 2, 50%) or similar level (*n* = 2, 50%) of dyspnea and fatigue in VRGs
Del Corral, Cebrià I Iranzo, López-de-Uralde-Villanueva, Martínez-Alejos R, Blanco, and Vilaró (2018) [19]	6MWT distance, MSWD, HJT, MBT, HG	Common muscle stiffness	Unsupervised and long follow-up period increases drop-out rate and nonadherence to exercise recommendations Using field tests instead of laboratory tests as assessments	VRG demonstrated improvement in all outcome measures (effect size: 0.25 to 0.85, *p* < 0.05) VRG demonstrated significantly greater improvement in all outcome measures (effect size: 0.99 to 1.96, *p* < 0.05) than CG
				12 months follow up: VRG showed a better MSWD than pre VRG (effect size: 0.29, *p* < 0.01) and a greater improvement than follow-up CG (effect size: 0.74, *p* < 0.05) VRG showed a better MBT, right HG, and left HG than pre VRG (effect size: 0.54, 1.08, and 0.88 respectively, all *p* < 0.01) VRG showed a better right and left HG improvements (effect size: 1.54 and 1.51, *p* < 0.01) than CG
de Corral, Percegona, Seborga, Rabinovich, and Vilaró (2014) [11]	HR, dyspnea, Fatigue, SpO_2_,	No adverse event	Lack of an incremental test to use as comparator Short duration of intervention session	Wii Active and Wii family Trainer VRGs achieved a higher % of predicted MHR (80.1 ± 7.4 and 82.1 ± 7.5 vs 79.8 ± 7.7 bpm, *p* < 0.01) than 6MWT No significant differences were found in SpO_2_ and dyspnea between all VRGs and CG Wii fit VRG showed a lower fatigue score (1.0 ± 1.3 vs 2.8 ± 2.5, *p* < 0.01) than CG
Frade, Dos Reis, Basso-Vanelli, Brandão, and Jamami (2019) [24]	Dyspnea, fatigue, SpO_2_, MHR, VO2 peak, number of steps in STVR	Not stated	Convenience recruitment of sample Lack of representation of population group No screening of function impairment which may affect gait No measurement of participant’s step length	VRG has a higher SpO_2_ (88.5 vs 85%, *p* < 0.05) and VO2 peak (13.5 ± 3.3 vs 12.6 ± 3 mL/min/kg, *p* < 0.05) than CG No significant differences were found in MHR, dyspnea, and fatigue score between VRG and CG. Good intra- and inter-rater reliability in VO2 peak (0.80 and 0.57 ICC, *p* < 0.001) and number of steps in STVR (0.94 and 0.93, *p* < 0.001)
Gomes, Carvalho, Peixoto-Souza, Teixeira-Carvalho, Mendonça, and Stirbulov (2015) [7]	HR, energy expenditure, treadmill distance and time, lung function	Not stated	Possible underestimation of energy expenditure with the chosen tool No individualized exercise intensity in the intervention group	VRG showed improvements in all outcome measures (size effect: 0.3 to 1.07, all *p* < 0.05), as well as CG (except for resting HR) VRG showed a higher predicted % of MHR than CG (103.2 ± 8.6 vs 96 ± 7.8, *p* < 0.05) VRG showed a higher total energy expenditure than CG (159 ± 41.6 vs 133.3 ± 32.1 calories, *p* < 0.05) CG showed a higher treadmill distance (895.8 ± 143.4 vs 703.3 ± 148.3 m, *p* < 0.05) than VRG
Holmes, Wood, Jenkins, Winship, Lunt, and Bostock (2013) [9]	HR, SpO_2_, dyspnea, and RPE	No adverse event	No objective measures of exercise intensity Replacing a laboratory treadmill test with a cycle ergometer, i.e. invalid measurement Small sample size	Exercise with VR showed an 86% of MHR demonstrated in CPET Less desaturation (*p* < 0.05) was evident during exercise with VR, comparing to CPET Lower dyspnea and RPE score (*p* < 0.05) were evident during exercise with VR, comparing to CPET
Kuys, Hall, Peasey, Wood, Cobb, and Bell (2011) [21]	HR, energy expenditure, SpO_2_, enjoyment, dyspnea, and fatigue	Not stated	No long-term effect examined Possible inaccurate measurement of energy expenditure with the armband design of energy expenditure measurement tool	VRG had a higher total energy expenditure (127 ± 55 vs 101 ± 55 kcal, *p* < 0.05) than CG VRG and CG showed a similar average HR (144 ± 13 vs 141 ± 15 bpm) during exercise VRG had a higher enjoyment score (7.3 ± 1.6 vs 4.7 ± 2, *p* < 0.05) than CG No significant difference in dyspnea (5.1 ± 2.1 vs 5.1 ± 2.2) and RPE (15.0 ± 2.6 vs 15.5 ± 2.6) were found between VRG and CG
LeGear, LeGear, Preradovic, Wilson, Kirkham, and Camp (2016) [22]	Total energy expenditure, HR, RPE, dyspnea, and SpO_2_	Not stated	Possible inaccurate measurement of energy expenditure with the armband design of energy expenditure measurement tool Small sample size	VRG showed a higher SpO_2_ (94.7 ± 2.5 vs 92.3 ± 3.3%, *p* < 0.0001) than CG No significant differences were found in total energy expenditure, HR, RPE, and dyspnea between VRG and CG
Liu, Meijer, Delbressine, Willems, Franssen, and Wouters (2016) [17]	6MWT distance, SpO_2_, HR, fatigue, and dyspnea	Not stated	CG performed one 6MWT and VRG performed two and the best attempt out of the two was chosen to analyze Time gap between GRAIL 6MWT and the post HR and SpO_2_ measurements Underrepresentation of GOLD stage 4 COPD patients and complex COPD patients in sample Monocentric study as limited access to the GRAIL Learning effect of GRAIL 6MWT was not established	Significant differences were found between VGS and CG in all outcome measures in over ground 6MWT (all *p* < 0.05) 6MWT distance: 511.0 ± 64.6 vs 668.8 ± 73.6 m Changes in pre- and post-SpO_2_: −7.1 ± 5.9 vs −1.2 ± 3.4% Changes in pre- and post-HR: 29.5 ± 11.8 vs 47.3 ± 15.7 bpm Changes in pre- and post-dyspnea: 4.0 ± 2.3 vs 1.1 ± 0.9 points Changes in pre- and post-fatigue: 3.7 ± 2.2 vs 1.1 ± 1.0 points Significant differences were found between VGS and CG in all outcome measures in GRAIL 6MWT (all *p* < 0.05) 6MWT distance: 483.7 ± 84.5 vs 692.3 ± 62.0 m Changes in pre- and post-SpO2: −2.0 ± 4.4 vs 0.0 ± 0.9% Changes in pre- and post-HR: 19.1 ± 10.5 vs 32.6 ± 15.1 bpm Changes in pre- and post-dyspnea: 3.4 ± 2.2 vs 1.0 ± 0.9 points Changes in pre- and post-fatigue: 3.2 ± 2.1 vs 1.1 ± 1.0 points
O’Donovan, Greally, Canny, McNally, and Hussey (2014) [25]	MHR, energy expenditure, VO_2_	No adverse event	Only recruited individuals with cystic fibrosis who were well and had a relatively good lung function Individuals require oxygen supplement were not recruited due to the requirement of wearing facemask during measurements	No significant differences were found in all outcome measures between VRGs and CG
Salonini, Gambazza, Meneghelli, Tridello, Sanguanini, and Cazzarolli (2015) [23]	HR, SpO_2_, dyspnea, and fatigue	Not stated	Only one short session of intervention, not enough to prove active gaming provides a sufficient training effect	Less participants in the VRG reached 80% of MHR (40 vs 67%, *p* < 0.05) than CG No significant between-group difference was found in SpO_2_ VRG experiences less fatigue and dyspnea (*p* ≤ 0.001) than CG
Sutanto, Makhabah, Aphridasari, Doewes, Suradi, and Ambrosino (2019) [20]	6MWT distance, dyspnea, QoL	Stated that all adverse events (including pulse rate higher than the predicted maximum, respiratory rate above 30/min, SpO_2_ below 90%) were recorded, but did not specify the event	Small sample size, underpowered No measurement of exercise intensity in VRG Standard exercise training may have masked the effect of the additional virtual reality gaming exercise Only the exercise component of traditional pulmonary rehabilitation was included No blinding applied to participants and assessors	Both VRG and CG demonstrated within-group improvement in 6MWT distance (52.4 ± 20.6, *p* < 0.0001 and 66.8 ± 27.8, p < 0.0001), and no between-group difference was found VRG showed a lower dyspnea score (4.5 ± 1.3 vs 5.7 ± 1.3, *p* < 0.05) than CG at baseline, and no difference was found between group after intervention Both VRG and CG demonstrated within-group improvement in health-related QoL (27.0 ± 14.3, *p* < 0.0001 and 24.6 ± 17.3, *p* < 0.0001), and no between-group difference was found
Simmich, Deacon, and Russell (2019) [13]	HR, SpO_2_, dyspnea, enjoyment	No studies reported the occurrence of adverse events linked to virtual reality gaming	Small number and significant heterogeneity of included studies Vulnerable to publication bias as only high-quality studies were included Possible language bias, only English search terms were used HKSJ estimation method may produce overestimation	No significant difference was found in HR, dyspnea, and SpO_2_ between VRGs and CGs after calculation of mean difference Large effect of enjoyment among VRGs was found comparing to CGs
Sánchez, Salmerón, López, Rubio, Torres, and Valenza (2019) [15]	Lung function, knowledge of condition, QoL, exercise capacity	Not stated	Small number of studies Heterogeneity of included studies	Knowledge of asthma were significantly improved in all educational VRGs Increase in exercise capacity, QoL, and improvement of symptoms were found in VRGs

*Abbreviations*: *VRG* virtual reality group, *CG* control group, *MHR* maximal heart rate, *HR* heart rate, *RPE* rating of perceived exertion, *6MWT* 6-min walking test, *SpO*_*2*_ oxygen saturation, *kcal* kilocalories, *bpm* beat per minute, *HJT* horizontal jump test, *MBT* medicine ball throw, *HG* hand grip, *MSWD* modified shuttle walk test distance, *STVR* stationary walk test with virtual reality, *GOLD* Global Initiative for Chronic Obstructive Lung Disease, *QoL* quality of life, *CPET* cardiopulmonary exercise test, *VO*_*2*_ oxygen consumption

### Assessment of methodological quality

All included articles were processed for the quality of analysis relevant to the research methodology. Differences in opinion were resolved by discussion or by a third reviewer (SG or CM). The Joanna Briggs Institute (JBI) critical appraisal tools [18, 27] were recognized as a reliable tool to investigate variations of study design including RCT, systematic review, and observational studies [28]. Outline of the results of the detailed analysis was created (Table 5).

**Table 5:** Quality analysis using Joanna Briggs Institute critical appraisal tools [18, 27, 29]

	**JBI Critical Appraisal Checklist for Systematic Reviews and Research Synthesis**													
**Author(s)**	**Q1**	**Q2**	**Q3**	**Q4**	**Q5**	**Q6**	**Q7**	**Q8**	**Q9**	**Q10**	**Q11**			**Total**
Butler et al. [6]	Y	Y	Y	Y	Y	Y	N	N	N	N	Y			7/11
Carbonera et al. [26]	Y	Y	Y	Y	Y	Y	N	N	Y	N	Y			8/11
Simmich et al. [13]	Y	Y	Y	Y	Y	N	N	Y	Y	N	Y			8/11
Sánchez et al. [15]	Y	Y	Y	N	Y	Y	U	N	N	Y	Y			7/11
	**JBI Critical Appraisal Checklist for Analytical Cross-sectional Studies**													
**Author(s)**	**Q1**	**Q2**	**Q3**	**Q4**	**Q5**	**Q6**	**Q7**	**Q8**	**Q9**	**Q10**	**Q11**	**Q12**	**Q13**	**Total**
Del Corral et al. [19]	Y	Y	N	N	Y	Y	Y	Y						6/8
Frade et al. [24]	Y	N	Y	Y	Y	N	U	Y						5/8
Holmes et al. [9]	Y	N	Y	Y	Y	N	Y	Y						6/8
Liu et al. [17]	Y	Y	Y	Y	N	N	Y	Y						6/8
O’Donovan et al. [25]	Y	Y	Y	Y	Y	N	Y	Y						7/8
	**JBI Critical Appraisal Checklist for Randomized Controlled Trials**													
Gomes et al. [7]	Y	Y	Y	N	N	Y	Y	Y	N	Y	N	Y	Y	9/13
Kuys et al. [21]	Y	Y	U	N	N	Y	Y	Y	Y	Y	Y	Y	Y	10/13
Del Corral et al. [11]	Y	Y	Y	N	N	Y	N	Y	Y	Y	Y	Y	Y	10/13
LeGear et al. [22]	Y	N	U	N	N	N	Y	Y	U	Y	Y	Y	N	6/13
Salonini et al. [23]	Y	Y	Y	N	N	Y	Y	Y	U	Y	Y	Y	Y	10/13
Sutanto et al. [20]	U	U	Y	N	N	N	Y	N	N	Y	Y	Y	Y	6/13

Key: *Y* yes, *N* no, *U* unknown

The interrater reliability for the observational and RCT indicated almost perfect agreement (*k* = 0.94) [30]. The interrater reliability for pilot studies indicated perfect agreement (*k* = 1) [30]. Most common problems encountered were randomization, assessor blinding, duration of study, and statistically significant population.

### Statistical analysis and synthesis

Meta-essential Workbook 3 (Version 1.5) [31] was used to perform a meta-analysis to investigate the effect on respiratory functions for the VR and VR/exergaming interventions compared with traditional exercises. RCTs were the only data included in the meta-analysis due to the high-quality study method. Effect sizes for independent continuous variables were calculated as standardized mean differences (SMD). SMD was used in cases where different methods across studies were used to assess the outcome measures because different types of VR and exergaming types were used across trials. The effect size was calculated as the difference in the outcome measure, reported at the end of trial from the control group and experimental group, where SMD ≥ 0.8 represented a large effect, 0.5-0.79 represented a moderate effect, and 0.2-0.49 a weak effect [32]. All standardized deviations were found within the included articles. Forest plots were completed on mean HR, peak HR, SpO_2_, and dyspnea on the difference between groups effect post-intervention. Articles were excluded from the meta-analysis where it was not comparable to a healthcare alternative.

## Results

### Description of studies

A total of 3766 studies were found through searches in PubMed, Cumulative Index to Nursing and Allied Health Literature (CINAHL), Embase, Medline, Web of Science, and Cochrane library. There were 22 articles that were included in this study and Table 1 outlines the selection process.

Out of these studies, three were conducted in Australia [9, 13, 21], three were conducted in Brazil [7, 24, 26], two in Canada [6, 22], one in Egypt [33], three in Italy [20, 23, 33], one in Ireland [25], one in the Netherlands [17], three in Spain [11, 15, 19], one in the UK [34], and four in the USA [35–38]. All eligible studies were published in English and were included in the quality analysis [27]. Of the 95 participants used in the meta-analysis, 49 (52%) were female and 46 (48%) were male.

The mean age and population in relation to conditions were analyzed from the literature. Six studies recruited individuals with CF (mean age 15.3 years, *n* = 189) [9, 11, 19, 21, 23, 26], one article analyzed asthma (mean age 7.75 years, *n* = 36) [7] and four articles analyzed subjects with COPD (mean age 64.96 years, *n* = 189) [17, 20, 22, 24]. Recruitment of participants was undertaken either by selection from an external health database [7, 11, 22, 24, 26, 39] or from inpatient programs [19, 21, 40, 41]. One study did not comment on recruitment methods [9], nine studies situated their data collection within outpatient settings [7, 9, 11, 17, 20, 22–25]. From these studies, six looked at the effects of VR/exergaming [7, 9, 11, 20, 23, 25], two analyzed the results of VR [22, 24] and one utilized augment reality [17], while only one article chose an inpatient setting to analyze effects with VR [21]. Lastly, one article researched the results of VR/exergaming intervention in the home setting [19].

All seven pilot studies were underpowered due to the small experimental groups (*n* ≥ 20). The pilot studies reporting mean age and population in relation to conditions are as follows: three pilot studies (mean age 67.75 years, *n* = 30) [34, 35, 39] discussed COPD, and one pilot study recruited subject with CF (mean age 9.3 years, *n* = 13) [36]. One pilot article reviewed a combination of conditions including COPD, bronchiectasis, interstitial lung disease, and asthma (mean age 71.2 years, *n* = 40) [33]. Whereas, Hoffmann et al. [37] investigated lung cancer (mean age 64.6 years, *n* = 7), and Yuen et al. [38] investigated idiopathic pulmonary fibrosis exclusively (mean age 69.8 years, *n* = 20), VR/exergaming was used as the experimental intervention in five pilots [33, 35, 37–39] and exergaming was investigated by two studies [34, 36]. Only one study used a comparator of video games instead of a traditional exercise program [38]. Home-based exercise interventions featured in five pilot studies [34–38], and two in inpatient [33, 39].

Qualitative results were collated from thirteen articles: one analytical cross-sectional study [19], five RCTs [7, 20–23] and seven pilot studies [33–39]. Two pilot studies [33, 38] were classified as RCT, according to JBI criteria. Quality analysis scores were completed, as described in the methods, as follows: The cross-sectional review was evaluated to be moderate to high quality 6/8 [19]. The average score for the RCT was 8.2/13. Three of the five RCTs achieved high-quality scores 9 </13; however, two articles [20, 22] reduced the average score. The lack of participant blinding, and randomization of groups contributed to their lower quality. Two pilot studies that were analyzed as RCTs [33, 38] were 5/13 and 11/13 respectively. The attributed difference in score was the absence of assessor blinding [33]. The pilot study score average was 5.4/9. The primary reasons for the relatively low score was ascribed to the fact that there were no designated control groups within the studies, and there was poor reliability of outcome measurements. The authors of this review focused on three qualitative findings to synthesize the results: adherence, enjoyment, and drop-out rate. Adherence was reported in five articles: four pilot studies [34, 37–39] and one cross-sectional review [19]. Enjoyment data was reviewed by four RCTs [20–23] and one cross-sectional [19]. Drop-out rate was reported by seven articles: two RCTs [7, 20], four pilot [33, 35, 36, 39], and one cross-sectional study [19].

### Synthesis

In the synthesis, 15 RCTs, observational studies, and seven pilot studies were analyzed. Seven critical considerations that illustrate the effect of VGS on individuals with a respiratory condition were taken into consideration, including mean heart rate, peak heart rate, SpO_2_, dyspnea, adherence, enjoyment, and drop-out rate. The meta-analysis only considers data from RCT studies.

Three qualitative findings were synthesized in this review: adherence, enjoyment, and drop-out rates. The aim was to understand if VR/exergaming exercise programs impacted these aspects of rehabilitation. Additionally, this was to determine if the intervention had a long-term viability in clinics and realistic use for patients with chronic conditions. The definitions and parameters for each qualitative finding varied among the studies. Adherence timeframe varied between 1 to 12 months. Studies that reported two data points [20, 39] observed significantly higher adherence at the first reporting over the final time point. Only one article [34] reported an increase in attendance rate through the testing period with the use of the digital rewards method. Since four out of five studies [19–23] reported statistically significant increased enjoyment from the VR/exergaming interventions over traditional rehabilitation, this may encourage further research with more standardized parameters because of its potentially positive influence on participants. Lastly, only seven [7, 19, 20, 33, 35, 36, 39] of the twenty-two studies reported in this review analyzed drop-out rates. This potentially conflicts with the findings for enjoyment. These articles found significantly higher drop-out rates for the intervention group. Few articles reported the reasons for drop-out. However, for articles that did report results, their findings were inconclusively linked to the intervention. The qualitative results were suggestive of positive VR/exergaming subjective participant experience. However, the lack of consistency between the studies made clear conclusions difficult.

### Critical considerations

#### Outcome measures

**Fig. 2:**
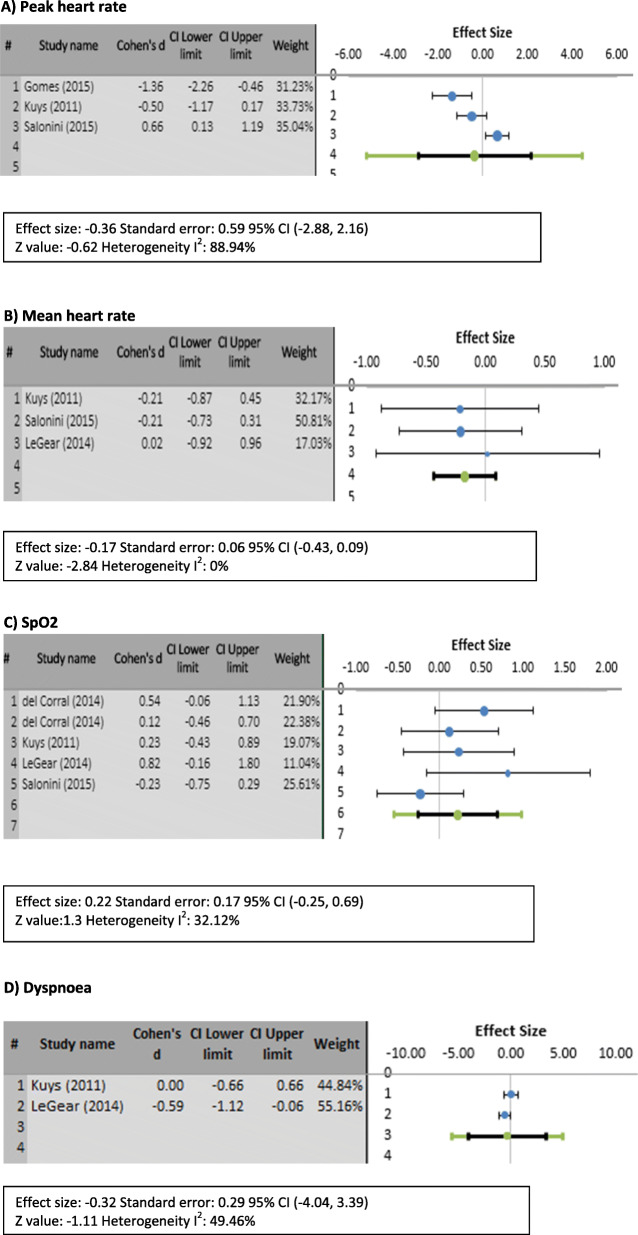
Forest plots demonstrating comparisons of outcome measures among included studies. **a** Peak heart rate. **b** Mean heart rate. **c** SpO2. **d** Dyspnea

Outcome measures used in the articles and pilot studies varied as shown in Fig. 2. Only the measures of mean heart rate, peak heart rate, SpO_2_, and dyspnea met the statistical criteria needed to perform a meta-analysis.

Peak heart rate was assessed by five studies [7, 17, 21, 23, 24]. Mean heart rate was measured by four studies [11, 20, 21, 23]. SpO_2_ was measured in five studies [11, 17, 20, 21, 24]. VO2 was analyzed in four studies [7, 11, 24, 25]. Dyspnea measured in five RCTs/observational studies [11, 17, 21–24] and four pilot studies [33, 36, 38, 39].

Duration was collated by total minutes of intervention use per experiment, six articles utilized the intervention for ≤ 30 min [11, 17, 21–24], while in three RCTs [7, 19, 20] and four pilot studies [33, 35, 37, 38], the total intervention time was greater than 60 min. Three different types of VGS (Nintendo Wii [11, 19–22, 25, 33, 35, 37–39], Microsoft Xbox [7, 9, 23, 24], and others [17, 34, 36] were used. Compliance was measured in only one RCT [19] and four pilot studies [34, 37–39]. Enjoyment was reported in five RCTs [19–23] and two pilot studies [37, 39]. Location types varied for these experiments. Nine studies [7, 9, 11, 17, 20, 22–25] looked at the effect of the intervention of subjects in an out-patient setting. One RCT [21] and two [33, 38] conducted their studies within in-patient settings. While one RCT [19] and four pilot studies [34, 35, 37, 38] investigated home-based intervention.

#### Peak heart rate

Three articles were analyzed for the effect size illustrated in Fig. 2a. Two articles [7, 21] found that the intervention did not achieve the HR peak average as compared with traditional exercise, with a SMD of −1.36 and 0.66 respectively. One article [23] showed a moderate effect of SMD 0.65. The average Cohen’s *D* (SMD −0.36; 95% [CI, −2.88-2.16] *p* = 0.27; *I*^2^ = 88.94%) indicated a weak effect of the intervention over traditional exercise.

#### Mean heart rate

Three articles were analyzed for the mean heart rate effect of the intervention compared with traditional exercise. Kuys et al. [21] and Salonini et al. [23] found a weak effect (SMD −0.21 [−0.87-0.45]) and one [22] found an insignificant effect (SMD 0.02 [−0.92-0.96]). Figure 2b presents the results of the meta-analysis in the format of forest plots. The average effect size reported as SMD −0.17 (95% [CI, −0.43-0.09] *p* = 0.002; *I*^2^ = 0%), indicating an insignificant effect of the intervention on mean HR as compared with traditional exercise.

#### SpO_2_

Four articles evaluated the intervention effect on SpO_2_. Del Corral et al. [11] is represented twice as they evaluated two different VRG (Wii Fit and Wii Active) with separate data as shown in Fig. 2c, with one (Wii Active) showed insignificant result (SMD 0.12; 95% [CI, −0.46-0.7]) and the other (Wii Fit) showed a moderate effect (SMD 0.54; 95% [CI, −0.06-1.13]). Two RCT studies [21, 23] reported a weak effect resulting in 0.23 [−0.43-0.89] and −0.23 [−0.75-0.29]. One study [40] reported SMD = 0.82 [−0.16-1.8], which represents a large effect size. The overall effect was weak (SMD = 0.26; 95% [CI, −0.25-0.69] *p* = 0.096; *I*^2^ = 66%).

#### Dyspnea

Two articles evaluated dyspnea using the BORG dyspnea scale as shown in Fig. 2d. One article [21] showed no effect (SMD 0 [−0.66-066]) and another [22] showed moderate effect size (SMD 0.59 [0.06-1.12]). The average Cohen’s *D* (SMD = 0.32; 95% [CI, −3.39-4.04] *p* = 0.13; *I*^2^ = 49.46%) indicated a weak effect of the intervention over traditional exercise.

#### Adherence

Adherence varied in the collection and reporting of data. The data collection methods varied: for example, self-reported (weekly phone call [19, 37], exercise log book [19, 34, 37], monthly e-mails [19], questionnaires [19, 38], mobile applications [34], direct supervision, with an in home visit [37], and supervision [39]. According to Hoffman et al. [37], adherence was calculated by the following equation: *number of intervention completed/number of interventions prescribed*, whereas Del Corral et al. [19] measured adherence as an average of 95% attendance per session at 6th week and 35% at 12th month. Burkow et al. [34] reported the additional use of digital rewards as a method to encourage adherence for home exercise programs, which is clinically relevant for determining cost-effectiveness. Burkow et al. [34] observed an average of 77% of participants reporting that digital rewards were influential to their attendance during the program. Leading to the average number of physical activity sessions per week was doubled from 2.9 (range 0-10, median 2) at baseline to 5.9 (range 3.3–10.33, median 4.8) during the testing period. Wardini et al. reported the mean adherence rate for the intervention group as 96.6 ± 3.4% individuals’ attendance per scheduled session [39]. Participants were deemed to be adherent with the program if they completed > 50% of sessions offered, using the equation: *sessions attended/sessions offered = attendance rate*. The authors report a 76% adherence rate, with a mean attendance rate of 64 ± 35% at the 6-week endpoint [39]. Yuen et al. [38] estimated adherence by the completion of a post-study survey. Attendance of participants that completed the prescribed interventions, with a frequency of three times per week for 30 min per day, was 20 ± 23%. However, the adherence rate rose to 42 ± 36% attendance when considering the recommended duration of 90 min per week for 12 weeks.

#### Enjoyment

Enjoyment was analyzed as a component of adherence by five articles. Four articles reported enjoyment in favor of the intervention, statistically significant [19, 21–23] utilizing different assessment methods: Likert scale [19, 22], 10-point analog scale [21], and survey [23]. Kuys et al. [21] provided graphical data while three articles [19, 20, 23] reported in percentage. One article [20] reported no statistical difference between the experimental and control groups, using the Saint George’s Respiratory Questionnaire. Only one pilot study looked at the enjoyment of the intervention, resulting in an average rating (5.56/6) using the Likert scale. Del Corral [19] is the only study to measure enjoyment at the duration of 12-months. The lack of heterogeneity of assessment tools used throughout these articles excluded the data points from the meta-analysis.

#### Drop-out rate

Drop-out was reported in three articles [7, 19, 20] and four pilot studies [33, 35, 36, 39]. Rates ranged from 5% [33] to 32% [36]. Six articles and pilot studies distinguished drop-out between the experimental and control group or had a crossover design [7, 19, 20, 33, 36]. The collective mean drop-out rate was 16% higher for the intervention groups compared with the control groups (73% and 57% respectively). The predominant reason for drop-out was no response including incomplete sessions [7, 19, 33, 35] accounting for 37% of drop-out reported in both experimental and control groups. Exacerbation of symptoms [20, 35], spontaneous rib fracture [35], and recurrent illness [34, 39] contributed to a mean average of 20% for the total drop-out. Of these, the articles that separated between experimental and control groups, exacerbation of symptoms [20, 35], were only reported for the intervention group. Only one study [39] reported drop-out due to participants regarding the intervention type too simplistic (*n* = 2) or difficult (*n* = 2), totaling four participants (57%) not included in the final analysis.

## Discussion

This systematic review and the subsequent meta-analysis contribute novel information by broadening the scope of the literature search to include well-designed pilot and observational studies. Also, this report synthesizes the results of traditional outcome measures and qualitative data. The purpose of this systematic review and meta-analysis was to examine the current evidence on the effectiveness of VR gaming and exergaming-based interventions for individuals with a respiratory condition. Heart rate (peak and mean), dyspnea, and respiratory function (SpO_2_ and VO2) were frequently reported to measure the effectiveness of exergaming and/or VR gaming intervention.

One of the main findings from this review is that exergaming-based interventions have been shown to produce an insignificant effect (SMD −0.17 [−0.43-0.09], compared with traditional rehabilitation) on mean HR. The difference can be potentially explained by the ambiguity in the indication of game intensity. The difficulty level of a video game is typically divided into levels (easy, moderate, and hard), which make it difficult to compare the intensity with both stationary bike and treadmill exercises. Moreover, the previous trial and review concluded that the HR response elicited by VGS achieves the recommendation intensity of training and will benefit the participants [9, 26]. A similar trend was observed in maximum HR (SMD −0.36 [−2.88-2.16]), which was comparable with previous findings [9, 26]. Salonini et al. [23] concluded a discontinuous HR trend with VRS and described gaming console exercise was similar to interval training with bursts of exercise followed by a short rest period. Although it resulted in a lower mean and maximum HR, the effect of high-intensity interval training is proven to be effective in improving compliance and cardiovascular fitness [23, 42].

Respiratory function is another frequently assessed outcome measure to determine the effectiveness of a rehabilitation exercise program. A weak effect was found in this meta-analysis of SpO_2_ (SMD 0.22, 95% [CI, −0.25-0.69]), as VRS resulted in a lower level of oxygen depletion and potentially less intense physical activity than traditional forms of exercise. Millet et al. [43] found that maximal oxygen consumption varied by exercise modalities, while specific training may have an effect on reducing oxygen consumption as a learning effect when the body develops an effective way to execute the movements.

VRS are comprised of whole-body exercises that have no fixed pattern and resemble daily activities including side-stepping (e.g., to maintain balance) and bend forward (e.g., to reach a target), in which people are well-trained. Potentially, this can be attributed to the incapability of VRS to reach maximal exercise intensity, as it was a type of whole-body exercise designed for entertainment with integral rest periods (game loading time). As indicated in a previous study, only maximal exercise was able to create a similar level of oxygen saturation when comparing treadmill exercise and stationary bike exercise [22]. There is an unaddressed research gap on comparing whole-body exercise to treadmill and stationary bike exercise which makes the comparison of VRS and traditional exercise challenging.

Easing of symptoms including shortness of breath is one of the compelling benefits of physical activity. However, dyspnea is also one of the common adverse effects resulting from intense physical activity, especially when the participants fail to control their symptoms with medication and an action plan [10, 44]. VRS exercise induced a noticeably lower level of dyspnea compared with traditional exercise. As VRS exercise is considered an interval training equivalent [23], the regular resting periods may influence to ease the symptom, yielding a lower dyspnea score [45]. According to de Jong and colleagues [46], fear of physical activity-triggered dyspnea causes avoidance of physical exercise and deconditioning in individuals with COPD. The result of the meta-analysis showed that VRG intervention point toward a reduction of a reported dyspnea score. This may encourage exercise participation among individuals with respiratory disease and improve their exercise compliance.

There is currently no definitive evidence to determine the impact of VGS programs on individuals with respiratory conditions.

However, the aim of this review was to guide the structure and focus of further studies. VGS as an intervention, as a compactor of a traditional exercise program, can increase enjoyment, reduce symptoms (dyspnea), and maintenance of cardiovascular fitness in an outpatient and home setting. Future qualitative and mixed method studies to explore various stakeholder perspectives and economic evaluations of the use of VRS within the management respiratory conditions would provide valuable insights for service development.

### Limitations

This review was limited by the heterogeneity of the studies and only six studies were included in the meta-analysis. A standardized value from the quality analysis could not be assigned to the articles, due to the lack of evidence and investigation of the JBI quality assessment tool. Enjoyment and adherence, which were crucial to the success of a pulmonary rehabilitation program, were not included in the meta-analysis because of the diverse outcome measures used [19–23, 37, 39]. Small sample size [9, 20, 22, 24] and biased target participants [17, 24, 25] may lead to biased findings, with similar difficulties reported in previous systematic reviews [6, 13].

## Conclusion

The results of this review illustrate that VRS can trigger physiological responses that benefit individuals with a range of respiratory conditions equal to that of a traditional exercise program. The use of VRS can provide options or adjuncts for rehabilitation, since the comparative results are equivalent to slightly diminished effect on heart rate, SpO_2_, VO2, dyspnea, and enjoyment. For those who only have access to a home program, VRS may be an effective and alternative method if initially supervised by a trained allied health professional. Adapting a VRS experience to focus on improving the respiratory outcomes and recovery of function for these individuals is a crucial factor for symptom reduction and quality of life. The field of VR/exergaming is dynamic; thus, it is essential that the most current and inclusive research guides clinical therapies.

## Data Availability

The datasets used and/or analyzed during the current study are available from the corresponding author on reasonable request.

## Abbreviations

VR: Virtual reality
VRS: Virtual reality systems
COPD: Chronic obstructive pulmonary disease
CF: Cystic fibrosis
VRG: Virtual reality group
AR: Augmented reality
MR: Mixed reality
CINAHL: Cumulative Index to Nursing and Allied Health Literature
CG: Control group
MHR: Maximal heart rate
HR: Heart rate
RPE: Rating of perceived exertion
6MWT: 6-min walking test
SpO^2^: Oxygen saturation
HJT: Horizontal jump test
MBT: Medicine ball throw
HG: Hand grip
MSWD: Modified shuttle walk test distance
STVR: Stationary walk test with virtual reality
GOLD: Global Initiative for Chronic Obstructive Lung Disease
QoL: Quality of life
CPET: Cardiopulmonary exercise test
VO^2^: Oxygen consumption
JBI: Joanna Briggs Institute
SMD: Standardized mean difference
